# An in situ approach to detect tree root ecology: linking ground-penetrating radar imaging to isotope-derived water acquisition zones

**DOI:** 10.1002/ece3.543

**Published:** 2013-04-10

**Authors:** Marney E Isaac, Luke C N Anglaaere

**Affiliations:** 1 Department of Physical and Environmental Sciences, University of Toronto Scarborough1265 Military Trail, Toronto, Ontario, Canada, M1C 1A4; 2 Department of Geography, University of TorontoOntario, Canada; 3 Forestry Research Institute of GhanaKumasi, Ghana

**Keywords:** Agroforestry, cocoa, edaphic conditions, Ghana, ground-penetrating radar, oxygen isotopes, root function, *Theobroma cacao*, tree physiology

## Abstract

Tree root distribution and activity are determinants of belowground competition. However, studying root response to environmental and management conditions remains logistically challenging. Methodologically, nondestructive in situ tree root ecology analysis has lagged. In this study, we tested a nondestructive approach to determine tree coarse root architecture and function of a perennial tree crop, *Theobroma cacao* L*.,* at two edaphically contrasting sites (sandstone and phyllite–granite derived soils) in Ghana, West Africa. We detected coarse root vertical distribution using ground-penetrating radar and root activity via soil water acquisition using isotopic matching of δ^18^O plant and soil signatures. Coarse roots were detected to a depth of 50 cm, however, intraspecifc coarse root vertical distribution was modified by edaphic conditions. Soil δ^18^O isotopic signature declined with depth, providing conditions for plant–soil δ^18^O isotopic matching. This pattern held only under sandstone conditions where water acquisition zones were identifiably narrow in the 10–20 cm depth but broader under phyllite–granite conditions, presumably due to resource patchiness. Detected coarse root count by depth and measured fine root density were strongly correlated as were detected coarse root count and identified water acquisition zones, thus validating root detection capability of ground-penetrating radar, but exclusively on sandstone soils. This approach was able to characterize trends between intraspecific root architecture and edaphic-dependent resource availability, however, limited by site conditions. This study successfully demonstrates a new approach for in situ root studies that moves beyond invasive point sampling to nondestructive detection of root architecture and function. We discuss the transfer of such an approach to answer root ecology questions in various tree-based landscapes.

## Introduction

A great deal is known about aboveground morphological and functional traits of plants in terms of (a) interspecific variation, (b) intraspecific plasticity, and (c) how these traits scale-up to influence community-level processes (Tilman 1990; Reich et al. 1997; Niinemets 2001). However, information is limited regarding such belowground traits, in particular, the knowledge gap on tree root response to the rooting environment with direct in-field measurement. Root distribution is an especially important measure of belowground competition; it is the plant architectural feature identifying soil resource horizon locations that are accessible to individual plants (Hodge 2004; Malamy 2005; De Kroon 2007). Moreover, as tree roots reportedly account for upwards of 40% of total biomass, forest tree roots hold a special position in carbon storage (Brunner and Godbold 2007).

Despite the importance of the tree root ecology, little is understood about in-situ root architecture and function until simple and nondestructive observational tools are developed that can be used to evaluate belowground patterns and processes (Pierret et al. 2005; Schroth et al. 2008; Zhu et al. 2011). Although a plethora of methods exist to quantify root dynamics in controlled conditions, there are a limited number of methods to measure tree root distribution in the field. Dominating these methods are point measurement with soil coring or destructive methods with root excavation, root growth with ingrowth cores and rhizotrones (Atkinson 2000; Smit et al. 2000). Empirical in situ confirmation of tree root response to environmental conditions has eluded ecologists due to the difficulties in sampling. Here, we sought to contribute to the development of such methods. Specifically, our study was designed to assess the potential for marrying newly developed tree root imaging technology with ground-penetrating radar (GPR) to isotopic analyses used to identify soil resource acquisition zones. The approach presented here allows for not only the determination of root architectural features, but also an indication of function, that is, water acquisition. Moreover, we are interested in whether this dual method can detect environment-induced modification to root systems. Managed perennial tree crop systems are an ideal place to develop new methods for understudied tree root ecology, as these systems represent a reasonable study point outside the complexity in forest systems.

### Measurement techniques: GPR and oxygen isotope analysis

GPR emits electromagnetic (EM) pulses into the ground at a known interval. Each EM pulse is altered by the dielectric permittivity of the subsurface materials. The amplitude and travel time of the reflected EM signals are recorded by the GPR's receiving antenna. EM waves are reflected at the soil–root interface due to a dielectric difference, and evidence supports a correlation between reflection patterns detected by GPR and tree roots in both controlled and field experiments (Butnor et al. 2001; Barton and Montagu 2004; Hirano et al. 2009; Cui et al. 2011; Hirano et al. 2012; Guo et al. 2013). Tree roots at a right angle to a GPR transect result in hyperbola-shaped response in the radar profile or slightly elongated hyperbola response if the transect is off the right angle (Butnor et al. 2001; Barton and Montagu 2004). Tree roots even at varying depths typically detected with GPR are >0.5 cm diameter (Butnor et al. 2003; Cui et al. 2011). Optimization of root detection has been reported in sandy soils, whereas higher clay context can drastically limit signal penetration (Butnor et al. 2003; Guo et al. 2013); propagation of GPR signals are highly dependent on soil electrical conductivity and dielectric permittivity (Conyers 2004). When conditions are favorable, GPR can be a powerful tool to determine root distribution and is less invasive and has a high resolution in contrast to classical methods of destructive, single-point measurements for inspecting stresses in trees. Recent study with GPR has advanced to biomass calculations based on processed radargrams and measured root data (Hirano et al. 2012; Guo et al. 2013).

Although GPR can clearly provide data on coarse root distribution, this technique is limited in its ability to provide information on root activity, for instance, root water and nutrient acquisition. Analysis of naturally occurring differences in isotopic enrichment of ^18^O in soils and plant tissue has been used to provide a nondestructive method to assess depth of tree water uptake zones (Ehleringer and Dawson 1992; Asbjornsen et al. 2008; Schwendenmann et al. 2010; Bertrand et al. 2012). Oxygen isotopic signatures in a soil profile can exhibit differences attributed to infiltration rates, plant water uptake rates, the degree of evaporative isotopic fractionation under the different vegetative covers, and processes of hydraulic redistribution (Ehleringer and Dawson 1992; Asbjornsen et al. 2008). As water is not isotopically fractionated during the uptake process in plant roots, plant tissue water isotopic signature is the same as the source water exclusively for preevaporative plant water, that is, xylem water (Dawson and Ehleringer 1993). Levels of extracted ^18^O in xylem water derived from nonphotosynthetic tissue samples of tree species can be matched to δ^18^O in water extracted from soil horizons below an individual tree, providing a natural marker of water acquisition zones (Brunel et al. 1995).

In this article, we present a new approach to detect tree root architecture and function in two edaphic environments. The objective is to chart the efficacy of this novel noninvasive in situ approach to determine tree coarse root distribution via geo-imagery and estimate root activity via water uptake. Moreover, we propose to use such active root zones to validate detected tree root distributions. We use GPR to determine root frequency distribution with depth and stable isotopic signatures of δ^18^O in plants and soils to determine active root zones via water acquisition. Employing plantations of the economically important tree crop *Theobroma cacao* L. (cocoa), this study was conducted under dominate edaphic conditions from two ecoregions of south-central Ghana, sandstone derived and phyllite–granite derived soils representing not only a range of conditions for GPR, but also a gradient to test the detection capabilities of root response to environmental conditions. It is hypothesized that (i) coarse root vertical distribution can be identified with GPR images and edaphic modifications to such root distribution will be detected, (ii) water acquisition zones can be estimated with δ^18^O plant and soil signature matching, and (iii) coarse root distribution will be correlated with measured fine root density and soil water acquisition zones. Our findings on root systems provide an advanced technique for nondestructive belowground studies. This will allow for further understanding of root ecology, particularly complementary resource partitioning in a range of tree-based landscapes.

## Materials and Methods

### Site description

The study was carried out in 2011, at two sites. The first was in South Formangsu, on a field research station operated by the Forestry Research Institute of Ghana (FORIG). The second, a shade-cocoa research farm of the University of Education, Mampong Campus, in Asante Mampong. South Formangsu and Mampong are located in the Asante Akim South and Sekyere West Districts of Ghana, respectively. Formangsu is situated between 6^°^23′ and 6^°^41′N and 0^°^56′ and 1^°^28′W, in the south-eastern part of the Ashanti region, Mampong is located between 6^°^55′–7^°^33′N and 0^°^55′–1^°^30′W, in the northern part of Ashanti region.

Formangsu in the Asante Akim South District lies within the Eastern Guinean forest ecoregion characterized by a moist semideciduous forest zone while Mampong lies on the border of this zone and the Guinean forest-savannah ecoregion characterized by a dry semideciduous forest zone. Both ecological zones are marked by double maxima rainfall. Mean annual rainfall ranges between 1500 mm and 1850 mm (Anglaaere et al. 2011). The Formangsu and the Mampong sites were both established on land that was previously secondary forest, cleared for food crop cultivation and then left to fallow for about 14 years. After clearance in 2001, sites were divided into blocks of 24 × 24 m and hybrid cocoa seedlings were planted at a regular spacing of ∼3 × 3 m.

### Site edaphic conditions

Soils in Formangsu fall within the Juaso-Morso Association covered by varied types of rocks mainly from Dahomeyan and Birrimean origin consisting of phyllite–granites and Tarkwaian sandstones over which the soils are developed. This has resulted in various soil associations encountered within the district. The texture of this soil association is generally medium, highly to moderately gravely, or deep and nongravely, and well to moderately well drained. The geology of the Sekyere West district (where Mampong is located), on the other hand, is made up of Upper Voltaian series mainly of sandstone, shale, and mud stone. Savannah ochrosols are found in the northern and eastern parts while forest ochrosols are found mostly in the southern and western parts. The soil is well drained, lateritic in nature, and moderately fertile as it is developed from Precambrian rocks of Birimian formation (Adu 1992; Adu and Mensah-Ansah 1995; SWDA 2004). Therefore, there are clearly two classes of edaphic conditions, sandstone site (Mampong site) and *phyllite*–*granite* site (Formangsu site).

Soil cores (*n* = 3) at 0–10, 10–20, 20–30, 30–40, and 40–50 cm depths were collected at the two sites to confirm soil textural class. Samples were dried in an oven for 48 h at 110°C and textural classes were determined with the hydrometer method. The sandstone site had significantly higher (F_4,8_ = 10.66; *P* = 0.0027) sand content in the top 20 cm with a sand percentage of ∼62.5% (Table 1). However, below 20 cm, the textural class shifted to a clay loam with an increasing clay fraction (F_4,8_ = 64.86; *P* < 0.0001) ranging from 26.7 ± 3.6% to 29.4 ± 1.3% (Table 1). The phyllite–granite site exhibited soils with a loam to silt loam classification, consisting of a significantly lower sand content ranging from 45.5 ± 5.5% to 53.0 ± 8.3% (F_1,4_ = 20.89; *P* = 0.0103) as compared with the sandstone site (Table 1).

**Table 1:** Mean (±SE) site characteristics (textural class distribution [percent sand, silt, and clay] and percent soil moisture content) with depth for the sandstone and phyllite–granite sites (*n* = 3)

Depth (cm)	Sandstone			Phyllite–granite		
	
	Sand (%)	Silt (%)	Clay (%)	Sand (%)	Silt (%)	Clay (%)
0–10	62.2 ± 5.9a	29.8 ± 4.5a	8.1 ± 2.3a	53.0 ± 8.3a	41.0 ± 6.3a	6.1 ± 2.0a
10–20	62.5 ± 4.7a	36.0 ± 6.5a	9.4 ± 3.5ab	46.2 ± 8.1a	41.7 ± 4.1a	12.1 ± 4.0ab
20–30	57.7 ± 3.3ab	27.6 ± 2.1a	14.7 ± 2.7b	45.7 ± 4.7a	40.3 ± 2.8a	14.1 ± 2.0b
30–40	50.6 ± 3.5bc	22.7 ± 1.6a	26.7 ± 3.5c	45.8 ± 3.9a	36.1 ± 1.9a	18.1 ± 2.0b
40–50	46.8 ± 3.1c	23.7 ± 2.0a	29.4 ± 1.3c	45.5 ± 5.5a	37.0 ± 2.0a	17.0 ± 1.0b

Means followed by the same letter in a column are not significantly different according to a Tukey's honestly significant difference test (*P* < 0.05).

### GPR data collection and interpretation

**Figure 1:**
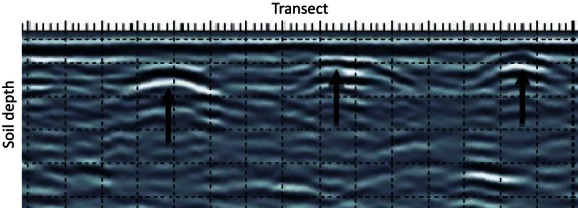
Example of a raw radar profile showing a 1.5 m detection transect to a depth of 0.5 m with grid marks at every 10 cm on the vertical and on the horizontal plane. Three root-induced hyperbolic-shaped reflections are shown with an arrow.

Using a 1 GHz GPR system (Noggin plus; Sensors and Software Inc. Mississauga, Ontario, Canada), detection transects on the edge of a 2 × 2-m grid centered on 10-year-old cocoa plants were scanned at the sandstone and phyllite–granite sites. Therefore, a total of four 2 m long detection transects 1 m from the cocoa stem were scanned for three distinct cocoa plants per site (*n* = 12 transects per site). During scanning, randomly identified hyperbolic reflections were selected and validated as roots with small-scale excavations. Raw GPR reflection data in detection transects were processed using velocity analysis as well as noise reduction, with the removal of low frequency through signal saturation correction, as well as amplitude compensation in EKKO Interp software (Sensors and Software Inc.). Soil velocity was accurately determined by inserting a metal rod at a known depth at each grid and using the GPR to determine the predicted depth and adjusted the set soil velocity accordingly. Subsurface images were visually inspected after image processing to identify tree roots via root-induced hyperbolas that cross through the transect plan (Fig. 1) and these hyperbolas were selected on radar profiles using EKKO Interp software (Sensors and Software Inc.). Coarse root counts along the detection transects were collapsed into soil profile depths of 10 cm intervals to a 50 cm depth and frequency distributions created as a percent of total counts per depth.

### Fine root data collection

A soil monolith of 0.5 m deep and 0.5 m wide was excavated on one edge of the grid after collecting GPR images (1 m away from the selected cocoa tree stems) (*n* = 3 per site). Soil cores (h = 5 cm, d = 5 cm) were taken at depths of 0–10, 10–20, 20–30, 30–40, and 40–50 cm and soils were washed on a 2-mm sieve to collect total fine roots in each core, which were dried and weighed. The samples were stored in plastic bags at 5°C and processed within 2 weeks. Samples were washed and cleaned of soil residue and fine root (<2 mm diameter determined with digital calipers) fragments were collected by hand with tweezers. Fine root biomass was expressed as dry matter per volume (100 cm^3^) per soil depth interval.

### Isotopic analysis

Another set of soil samples (*n* = 3 per site) were collected on another edge of the grid (1 m away from the select cocoa tree stems) with an auger to depths of 0–10, 10–20, 20–30, 30–40, and 40–50 cm in order to create a soil water isotope abundance profile for each site. Samples of nonphotosynthetic core tissue of the cocoa neighboring the soil sampling location were collected with a tree corer (*n* = 3 per site). All plant and soil samples were placed immediately in scintillation vials after collection, sealed with parafilm to prevent evaporative fractionation prior to vacuum distillation, and refrigerated. Plant and soil samples for each block at each site were consistently collected on the same day to avoid shifts in isotopic signature patterns due to seasonality (Asbjornsen et al. 2008). All samples were collected within 2 weeks before the onset of the rainy season.

Plant tissue and soil samples were extracted in a vacuum distillation line (Ehleringer and Osmond 1989; West et al. 2006) and analyzed with a Picarro H_2_O Cavity Ring-Down Spectrometer model L1102i (Picarro, Santa Clara, CA) for d18O isotopic composition (Laboratory for Stable Isotope Sciences, Western University, Ontario, Canada). δ^18^O was calculated as:


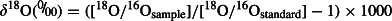


using Vienna Standard Mean Ocean Water (VSMOW) as the standard (Dawson 1993) with a precision of 0.2‰. The δ^18^O signature in plants was compared with the δ^18^O signature in the soil profile to characterize potential water uptake zones (Brunel et al. 1995). Although limitations with this approach have been identified (Asbjornsen et al. 2007, 2008) for instance irregularities in the soil profile isotopic gradient, identifiable soil water uptake depths are predictable when conditions are suitable for uniform soil isotopic gradients.

### Statistical analysis

Analysis of variance (ANOVA) using the general linear model (PROC GLM) procedure in SAS was conducted on the effects of soil depth on soil textural class, measured fine root density, and soil δ^18^O signatures. Independence, randomness of residuals, and a mean error equal to zero were confirmed with a test of residuals. Normality of residuals was tested using the Shaprio–Wilk test. Measured fine root density data were square root transformed. As detected coarse root count data did not meet normal distribution assumptions, a nonparametric Kruskal–Wallis test was conducted on the effects of soil depth on detected coarse root count as a percentage of total count data. Significant ANOVA's were tested with Tukeys' honestly significant difference test. Linear correlations between detected coarse root count and measured fine root density and between detected coarse root counts as a percentage of total counts and the percent difference in soil δ^18^O to the identified water acquisition zone were conducted with PROC CORR procedure in SAS. All statistical analysis was conducted in SAS version 9.2 (SAS Institute, Cary, NC) with a significance level set at *P* = 0.05 for all tests.

## Results

### Detected coarse root distribution

**Figure 2:**
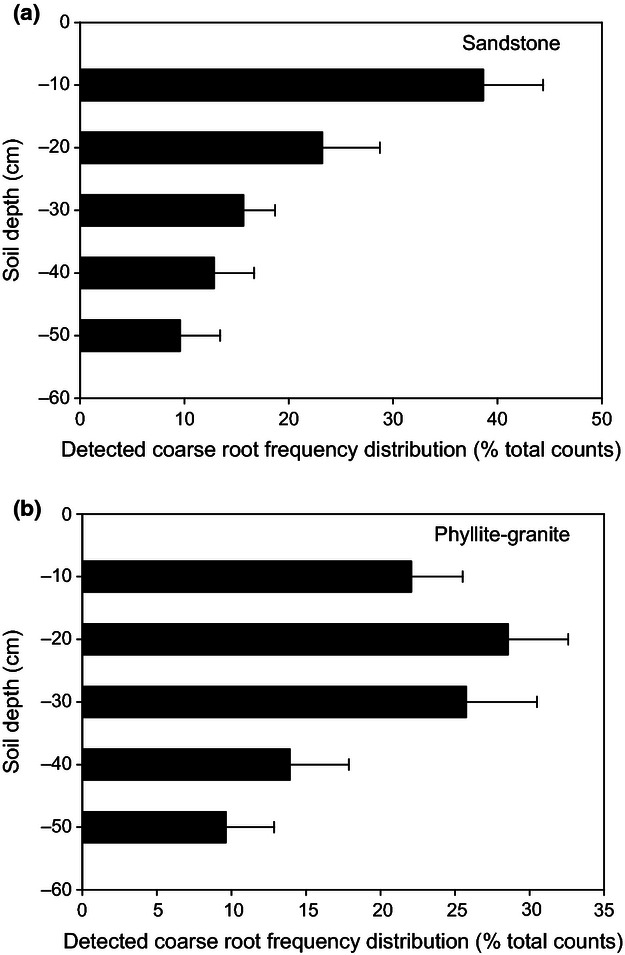
Mean detected coarse root frequency distribution as a percentage of total counts with depth based on root-induced hyperbolas identified in ground-penetrating radar profiles at a 1 m distance from a cocoa tree for the (a) sandstone and (b) phyllite–granite site. Bars represent ± SE of the mean (*n =* 12).

Detected coarse root count as a percentage of total counts significantly varied with depth at the sandstone (*P* = 0.0013) and phyllite–granite (*P* = 0.0059) sites (Fig. 2a and b). Subsurface image analysis revealed higher coarse root frequency (38.6 ± 5.7%) in the top 10 cm soil depth then declining by 40% in the 10–20 cm depth at the sandstone site (Fig. 2A). Detected coarse roots at the phyllite–granite site were more evenly distributed in the top 30 cm with a decrease in frequency below 40 cm as compared with the top of the soil profile (Fig. 2b).

### Measured fine root density

**Figure 3:**
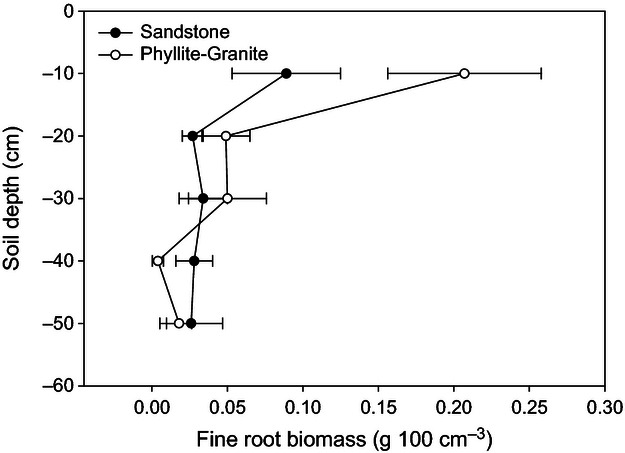
Mean fine root density (g 100 cm^3^) at the sandstone and phyllite–granite sites for cocoa in monoculture. Bars represent ± SE of the mean (*n =* 3).

**Figure 4:**
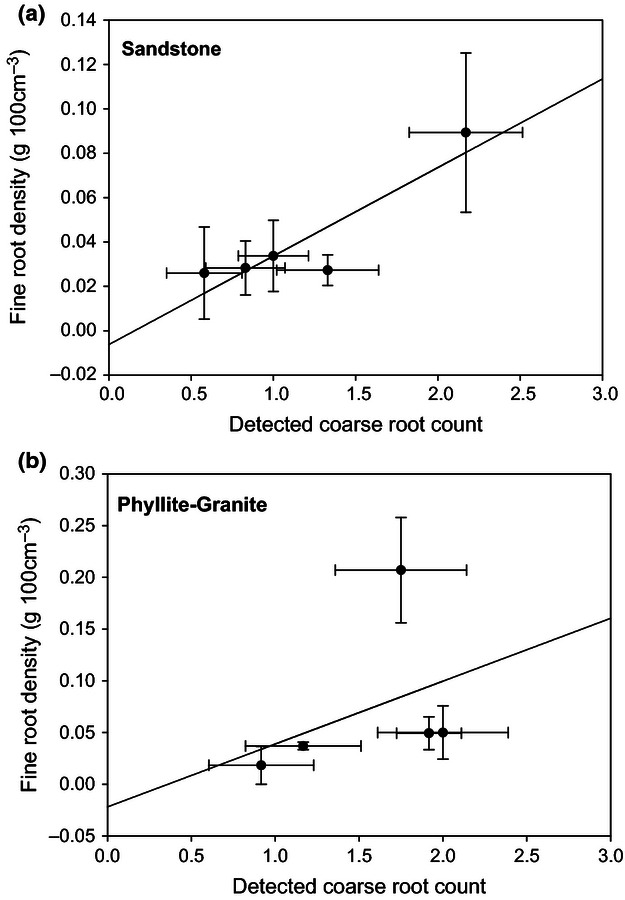
Mean fine root distribution (g 100 cm^3^) in relation to mean detected coarse root count based on root-induced hyperbolas identified in radar profiles for the (a) sandstone (*r* = 0.90; *n* = 5; *P =* 0.0357) and (b) phyllite–granite (*r* = 0.38; *n =* 5; *P =* 0.5252) site. Bars represent ± SE of the mean (*n =* 3 for fine root density; *n =* 12 for detected coarse root count).

Mean fine root density at a distance of 1 m from the cocoa tree stem was significantly higher (F_4,8_ = 7.29; *P* = 0.0089) in the top 10 cm depth as compared with any of the lower depths in the soil profile at the phyllite–granite site (Fig. 3). However, at the sandstone site, no significant difference was found in fine root distribution, although measured values did decline with depth (Fig. 3). A significant positive linear correlation between mean detected coarse root counts and measured fine root density was found for the sandstone site (*r* = 0.90; *n* = 5; *P* = 0.0357; Fig. 4a) indicating that higher coarse root frequency is linked to higher fine root biomass. On the contrary, fine root density was not significantly related to mean detected coarse root counts at the phyllite–granite site (*r* = 0.38; *n* = 5; *P* = 0.5252; Fig. 4b),

### Gradients in soil isotopic composition

**Figure 5:**
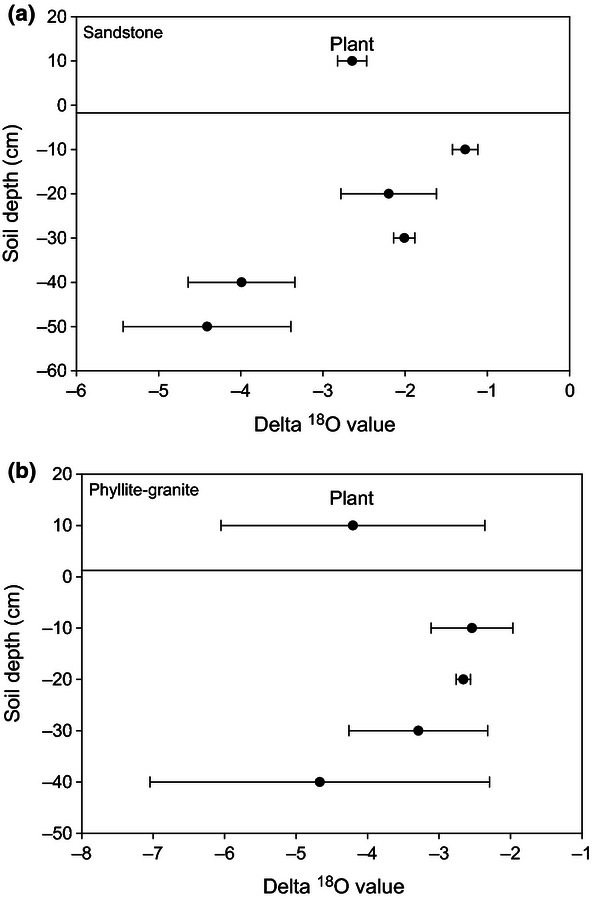
Mean δ^18^O values of soil water with depth and δ^18^O of plant tissue (Plant) at the (a) sandstone and (b) phyllite–granite site. Bars represent ± SE of the mean (*n =* 3).

At the sandstone site, a significant decrease (F_4,7_ = 4.33; *P* = 0.0446) in δ^18^O values occurred with soil depth, illustrating less negative isotopic values near the soil surface, presumably due to evaporation of the lighter ^16^O isotope, thus enriching the signature deeper in the profile (Fig. 5). Profile δ^18^O signatures at the phyllite–granite site were extremely variable thus masking any distinguishable profile shifts in δ^18^O (Fig. 5).

### Isotopic composition of plant water

At the sandstone, nonphotosynthetic plant tissue of cocoa site exhibited δ^18^O isotopic signatures ranging from −2.77‰ to −2.52‰ (Fig. 5a). Via plant–soil isotopic matching, the water acquisition zone was identified at the 10 to 20 cm depth. On the phyllite–granite site, cocoa exhibited a large range of δ^18^O isotopic values from −2.90‰ to −5.51‰, presumably due to highly variable soil δ^18^O isotopic values or mixing of soil water from multiple uptake zones (Fig. 5b), therefore no clearly identifiable water acquisition zone for cocoa was determined at this site.

**Figure 6:**
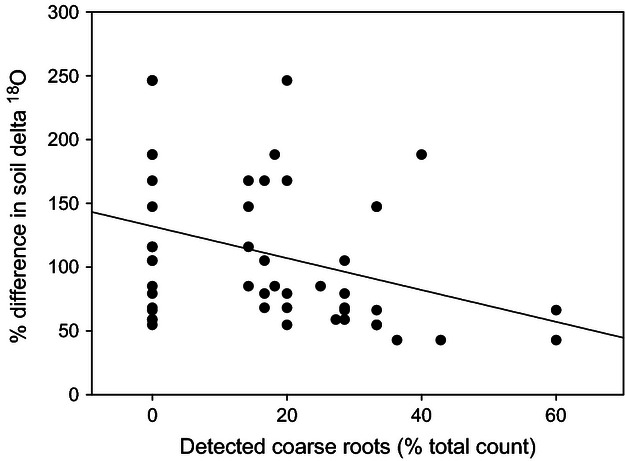
Correlation between detected coarse roots (% total count) and the percent difference in soil δ^18^O value of the corresponding root depth and the mean δ^18^O of the predicted uptake depth based on plant–soil matching at the sandstone site (*r* = −0.33; *n =* 51; *P =* 0.0205).

The percent difference between the soil δ^18^O isotopic value of a corresponding root depth and the mean soil δ^18^O of the predicted uptake depth based on plant–soil matching was correlated with coarse root distribution. At the sandstone site, these percent differences, in relation to the detected coarse root frequency, were significant (*r* = −0.33; *n* = 51; *P* = 0.0205; Fig. 6). This negative relationship shows that with greater coarse root frequency, the percent difference to the identified acquisition soil depth approached zero; a greater root frequency accurately indicates the isotopic derived water uptake zone. However, again, due to indistinguishable water acquisitions zones at the phyllite–granite due to inconsistent soil isotopic gradient, no relationship was determined for coarse roots and uptake at this site.

## Discussion

In situ detection of tree root ecophysiology is hindered by methodological challenges. Current techniques such as GPR for root detection and linked biomass estimates are rapidly advancing (Guo et al. 2013). However, there still remains a need for (1) progression from exclusively structural features of roots to functional features and (2) nondestructive validation of GPR detection. In this study, we proposed to achieve these two goals with identified water acquisition zones via water isotopes. By meaningfully adding a measure of root function, we advance this increasingly important method.

GPR profiles exhibited hyperbolic responses in all profiles indicating detectable roots to a depth of ∼0.5 m under both edaphic conditions. Our findings support previous evidence of tree root reflection patterns detected by GPR in both controlled and field experiments (Hruska et al. 1999; Butnor et al. 2001; Barton and Montagu 2004; Hirano et al. 2009, 2012; Cui et al. 2011). We illustrate a decrease in coarse root frequency with depth at the sandstone site as a percentage of total roots whereas the phyllite–granite site induced a clear increasing trend of coarse root counts to 30 cm depth (Fig. 2b). The depth of penetration and resolution of GPR has reportedly been dependent on soil composition particularly attenuation of EM waves on lower sand content soils (Butnor et al. 2001; Guo et al. 2013). However, we suggest that the variable vertical root distributions found in this study are not a product of poor wave resolution as depth of penetration was relatively shallow and amplitude compensation was applied to geo-images. Indeed, these root distributions demonstrate that the GPR detected edaphic effects on root ecologies; intraspecific plasticity was clearly demonstrated for detected root distribution between the two sites.

Our predicted water uptake zones, based on isotopic plant–soil matching, support these intraspecific root distribution findings. To achieve conditions for plant–soil matching, we expected that the gradient in soil δ^18^O in the soil profile would follow a clear decreasing pattern but this expectation was only half met; we found such a gradient but exclusively on the sandstone site. This confirms previous study suggesting strong environmental influences on natural abundance isotopic signatures in soil profiles (Asbjornsen et al. 2008) even under plantations of the same species as in our study. On the sandstone site, plant tissue exhibited an isotopic signature ranging from −2.77‰ to −2.52‰, much narrower than at the phyllite–granite site (−2.90‰ to −5.51‰). Using plant–soil matching, the plant isotope range on the sandstone site indicates a water acquisition zone at 10–20 cm soil depth. Similarly, with deuterium as a natural tracer to study water uptake patterns in the same species, Schwendenmann et al. (2010) showed that water uptake by cocoa occurred primarily in the top 30 cm horizon. Advantageously, on the sandstone site, we found a clear trend and suggest that the 10–20 cm soil depth is categorically the most active depth for water uptake. It is possible that trees on the phyllite–granite site are absorbing water over a range of soil depths, presumably due to resource patchiness or inconsistent availability by depth, creating a mixed signature in the xylem tissue, as suggested by Asbjornsen et al. (2007). Our detected coarse root distribution data supports this as a much larger root distribution zone on the phyllite–granite sites was detected (Fig. 2). Our findings from the two techniques in this study converge to depict similar belowground intraspecific plasticity.

Fine roots have long been shown to play a crucial role in nutrient and water absorption (Atkinson 2000; Cahill et al. 2010) and particularly for our test species, cocoa (Muñoz and Beer 2001; Moser et al. 2010). In our study, measured fine roots were highly concentrated in the top 10 cm depth at the phyllite–granite site, exhibiting a 76.2% reduction below this soil horizon and another sharp decline in fine roots below 40 cm. At the sandstone site, fine root distribution showed a 69.4% reduction below the top 10 cm interval, similar to detected coarse roots. Importantly, fine root density was positively correlated with detected coarse root count but exclusively at the sandstone site (Fig. 4a and b), suggesting that fine root distribution is not only dependent on edaphic conditions but is highly related on coarser root distribution under certain conditions.

In response to the limitations of GPR utility for tree root determination and the need for a root location validation technique, we use natural abundance water isotope tracers to validate GPR-detected root distributions. We show a significant positive relationship between detected coarse roots and active acquisition zones as determined by soil–plant isotopic matching at the sandstone site (Fig. 6) providing evidence for accurate GPR detected root locations. Our findings not only support this use of water isotopes as natural tracers of root activity, but this isotopic data also acts as a validation method of GPR root detection. This validation approach should be tested across other spatial or even temporal gradients, for instance, to capture ontogeny of root ecophysiology.

Advancing root ecology studies to reconcile the gap in our understanding of not only intraspecific but interspecific root ecophysiology is particularly important for root recognition and response (De Kroon 2007; Cahill et al. 2010). Literature suggests two dominant mechanisms for interspecific spatial complementarity are commonly identified in plant systems: morphological root plasticity in response to resource availability and/or the presence of competing plants, and existing independent root architecture in separate soil resource horizons (Hauggaard-Nielsen and Jensen 2005; De Kroon 2007; Hinsinger et al. 2009; Cahill et al. 2010). With species-specific plant δ^18^O isotopic signatures at the same site (Schwendenmann et al. 2010), we argue that GPR-detected roots can be noninvasively species differentiated in order to determine critical aspects of niche partitioning. Root studies have been limited by unavailable data on multispecies root performance in managed systems as well as root behavior to management change. Repositioning this approach to evaluate belowground dynamics, specifically in multispecies systems, is promising. For our test species cocoa, research under similar conditions has shown many beneficial aboveground response patterns to the integration of shade trees (Isaac et al. 2007; Isaac and Kimaro 2011) but determination of belowground root patterns to the presence of shade trees in these perennial cropping systems, particularly developmental response to management practices, is central to the long-term success of agroforestry production systems (Schroth et al. 2008; Moser et al. 2010).

To our knowledge, this is the first study that links tree root imaging with a measure of root activity in situ. We demonstrate that cocoa coarse root distribution can be determined nondestructively by geo-imagery and this distribution will be dependent on environmental conditions thus supporting our first hypothesis. Although coarse root properties are important to plant performance, we acknowledge that fine roots play a crucial role in soil resource adsorption and therefore link our detected coarse root distribution to measured fine root density, illustrating a strong positive correlation between coarse root and fine root distribution with depth but exclusively on sandstone sites. With the use of the oxygen isotopes, we estimate active acquisition zones with matched soil and plant δ^18^O signatures. Moreover, GPR-detected root distribution successfully indicated these active root zones. Although the approach we present here shows promise in providing both architectural and functional data on tree roots, limitations are evident. Most importantly, we rely on a strong relationship between coarse and fine root distributions. This was not achieved at our phyllite–granite site, indicating edaphic-based limitations for this approach. Further, soil isotopic gradients are required for plant–soil δ^18^O matching to be successful; again, our approach is limited to soils that achieve such gradients.

Empirical in situ confirmation of belowground dynamics has eluded ecologists due to the difficulties in data collection, however, the new approach presented here allows for not only the determination of root structure, but also an indication of function, that is, water acquisition. This is critical for appropriate management prescriptions of tree-based land use systems under various resource stresses.
